# Research and Experiment on the Self-Calibration Mechanism of the Position and Orientation of Micro-Component Based on Droplet Array

**DOI:** 10.3390/mi17060669

**Published:** 2026-05-28

**Authors:** Yan Hu, Qin Zhang, Yueshu He

**Affiliations:** School of Mechanical and Automotive Engineering, South China University of Technology, Guangzhou 510640, China; hyxyz1094@126.com (Y.H.); heyueshu@hotmail.com (Y.H.)

**Keywords:** droplet array, micro-component, self-calibration, mechanism

## Abstract

The self-calibration of micro-component position and orientation is a key step in micro-assembly. To address the limitations of conventional self-calibration methods—where the calibration substrate is fixed and lacks adaptability—this study proposes a droplet-array-based method for self-calibrating micro-component position and orientation. By using a droplet array to form a reconfigurable calibration substrate, the method supports iterative updates of micro-devices and enables synchronous restructuring of the substrate. First, a mechanical model of the self-calibration process is established to analyze the coupling forces exerted by the liquid-bridge array between the calibration substrate and the micro-component, thereby clarifying the mechanism of droplet-array-driven self-calibration. Next, the effects of micro-component material and surface properties on calibration error are examined. Extensive experiments are then conducted to validate the proposed analytical approach. The results show that a droplet array matching the shape and size of the micro-component can be constructed in real time as a calibration substrate. Through the coupling forces generated by the liquid bridges, self-calibration of micro-components with arbitrary shapes and dimensions can be achieved. Calibration accuracy is dependent upon the material and surface roughness of the micro-component. Variations in the micro-component material lead to different forces being applied by the liquid bridge, with the self-calibration error arising from the interplay of these factors. For micro-components of identical material, a smoother surface corresponds to higher calibration accuracy.

## 1. Introduction

Micro-component position and orientation self-calibration technology is a crucial step in micro-assembly, widely used in fields such as electronic manufacturing and biomedical applications. With the ever-accelerating pace of innovation in mechanical and electronic products, micro-components are exhibiting a trend toward higher precision, reduced thickness, and greater diversification. To accommodate the developmental requirements of these micro-components, the advancement of high-throughput and highly self-adaptive calibration techniques is a key future direction, a pursuit that has led numerous researchers globally to undertake substantial investigative work.

Currently, calibration methods for the position and orientation of micro-components are mainly divided into active calibration methods and passive calibration methods. Active calibration methods generally achieve closed-loop control through miniature multi-DOF manipulators or grippers, assisted by visual or force feedback. Kenta Tanaka et al. [1,2] developed a capillary force gripper with an automatic liquid supply function, and integrated with CCD cameras for top-view and front-view imaging, achieving the positioning and manipulation of complex-shaped micro-components. References [3,4,5] developed novel MEMS micro-grippers integrating grasping and releasing functions, achieving high-speed and reliable automatic positioning for micro-components with sizes of several micrometers through visual recognition, detection, and control. References [6,7] proposed an automatic alignment method and apparatus for micro-components based on hybrid visual servoing. By combining position-based and image-based visual servo control methods, this approach simplified the alignment process, improved efficiency, and realized the automatic alignment and assembly of micron-scale silicon arms and aluminum shells. Tang, Y. L. et al. [8] proposed a confocal co-imaging alignment micro-assembly system and calibration method, overcoming the optical diffraction limit in conventional microscopic optical observation and realizing the automatic alignment and assembly of millimeter-scale micro-components. Although the aforementioned active methods offer high precision, they typically rely on dedicated actuators, visual closed-loop systems, or multi-DOF platforms, making it difficult to simultaneously align multiple components or adapt to arbitrary geometric shapes.

Passive calibration methods generally adopt an open-loop approach. Essentially, this process involves calibrating a micro-component from a random initial position and orientation to a desired position and orientation under a specific driving force. Based on the driving mechanism, these methods can be classified into those driven by external forces and those based on fluid surface tension.

Self-calibration methods driven by external forces mainly rely on magnetic fields [9,10], dielectrophoresis and other approaches to realize calibration. Ramadan et al. [11,12,13] proposed a large-scale micro-component self-assembly method utilizing an external magnetic array, which combines magnetic attraction, shape recognition, and mechanical vibration to achieve high-throughput alignment and assembly of micro-components. In recent years, dielectrophoresis (DEP) has also been applied to the precise manipulation and assembly of microparticles, yet it generally requires sophisticated electrode arrays and specific dielectric environments [14]. Bashir’s team [15] introduced a silicon resistor fluid assembly method based on dielectrophoresis and electrohydrodynamics, achieving sub-micron precision alignment and assembly of silicon resistors and silicon blocks on microelectrode structures. Furthermore, recent studies on electrowetting [16,17], shape memory polymers [18] and acoustic fluidic control techniques [19] have provided new ideas for programmable droplet manipulation. However, their application potential in high-precision self-calibration of micron-scale micro-components has not been fully explored. However, these methods require external driving equipment, making the system relatively complex, and the effects of external forces (such as magnetic force and electrohydrodynamic forces) on micro-components remain unclear, limiting their widespread application in practice.

In the self-calibration method based on minimizing the surface tension energy of liquids [20,21,22,23,24,25,26,27,28,29,30,31], surface tension serves as an effective driving force for the self-calibration and self-assembly of micro-components. This method enables large-scale integration of micro-components on substrates and is widely used in self-assembly applications. Srinivasan et al. [32,33] utilized fluidic techniques based on hydrophobic SAMs (self-assembled monolayers), patterned shapes and capillary forces to construct various-shaped microstructures on silicon and quartz substrates, achieving self-assembly of micro-components with various shapes ranging from nanometers to micrometers in water. Reference [34] proposed an idea of low-temperature fluidic self-assembly and self-alignment of chips using core–shell transformation-imprinted solder bumps. This approach enables high-throughput self-calibration and self-assembly of chips, and it also analyzes the impact of different material ratios on self-calibration performance. Bo Chang et al. [35] sprayed micro-rain in the assembly area to form liquid bridges, inducing the microchips to move towards the substrate, achieving self-transport and self-assembly of millimeter and submillimeter-scale chips. Stauth et al. [36] studied the effects of capillary forces and fluid forces on the self-assembly of micro-components at binding sites and extended the self-assembly process to each substrate containing fixed binding sites, achieving high-throughput self-assembly of micro-components. C. Gary Tsai et al. [37] proposed a method for the self-alignment of microchips driven by both surface tension and solid edges in response to the demand for large-scale and low-cost packaging of microchips such as RFID and LED chips. By designing the physical structure of the substrate to replace complex surface chemical treatments, this method reduces costs while improving process reliability.

A comprehensive review of the current research, both domestically and internationally, reveals that self-calibration methods based on the principle of minimizing fluid surface tension energy are characterized by their simple structure and ease of implementation, leading to widespread applications in self-assembly. However, existing technologies typically require the fabrication of different calibration substrates based on the sizes and shapes of the micro-components. When the size, shape, and other parameters of the micro-components change, the substrate needs to be redesigned and manufactured, resulting in fixed calibration shapes and poor flexibility, making it difficult to meet the self-calibration needs of diverse micro-components. To address this issue, international scholars have proposed improved strategies such as dynamic template regulation and deformable substrates. Weinstein et al. [38] construct reversible capillary bridges using underwater bubble arrays. By regulating the quantity, arrangement and size of bubbles, they realize the grasping, releasing and directional arrangement of objects with diverse shapes and surface wettability, demonstrating the potential of liquid–gas interfaces as reconfigurable manipulation units. Constante et al. [39] fabricate lamellar structures with high aspect ratios via melt electro-writing technology. Combined with the thermal response characteristics of shape memory polymers, the surface morphology can be driven by the deformation of droplets themselves, thus achieving programmable regulation of wettability and adhesion force. Guo et al. [40] developed a photosensitive polymer stamp. Local adhesion switching and surface morphology modulation can be realized through masked ultraviolet exposure, enabling the programmable transfer and assembly of micro-components over a large area (4-inch scale) without sophisticated photolithography processes. Zhang et al. [41] directly ablated micro-grid structures on the surface of shape memory polymer stamps using nanosecond ultraviolet lasers. Combined with hot pressing and thermal recovery procedures, dual macroscale and microscale tunability of surface superhydrophobicity and adhesion force is achieved, and programmable micro-transfer printing on various substrates is accomplished. Jeske M P et al. [42] fabricated microstructured surfaces with self-complementary sinusoidal profiles via high-precision two-photon polymerization (2PP) 3D printing technology. Through the design of matched sinusoidal patterns, predictable and repeatable interfacial contact can be formed when two surfaces come into contact. Their work systematically investigates the effects of surface geometric parameters, including amplitude and wavelength, on adhesion force, and reveals the quantitative correlation between adhesion performance and surface morphology. Although the above methods enhance operational flexibility, they still essentially rely on partial modification of substrate physical deformation or surface chemical modification, and fail to fundamentally resolve the core scientific challenge of decoupling substrate characteristics from device parameters. To overcome the insufficient adaptability caused by the physical structure locking effect of conventional rigid substrates, the concept of flexible and reconfigurable calibration substrates emerges.

In recent years, the rapid development of digital microfluidic technologies has enabled the high-precision, programmable regulation of discrete droplet morphologies at the microscale, providing solid technical support for the aforementioned theoretical concepts [43,44,45,46,47,48]. In broader research on droplet microsystems, droplets are not only utilized as microreactors but are also designed as independent single-cell analysis platforms and micro-experimental environments with controllable interfacial properties, which are applied in studies such as high-throughput screening, cellular behavior regulation, and external stimulus responses [49,50,51,52,53,54]. For example, Brouzes et al. [49] employed droplets as microreactors for single-cell biochemical screening, achieving high-throughput toxicity testing of drug libraries; Raveshi et al. [50] constructed microenvironments with varying curvatures using droplets to investigate the regulatory effect of interfacial curvature on sperm motility; Vafaie et al. [51] implemented repetitive pulsed ultrasound stimulation on individual human sperm within droplets to explore non-invasive vitality maintenance methods. These studies demonstrate that the geometric characteristics, interfacial properties, and spatial distribution of droplets have become critical factors influencing system functionality. Furthermore, advancements in manipulation technologies, including droplet generation, splitting, detection, and sorting, have laid a solid foundation for constructing reconfigurable, function-oriented droplet systems [52,53,54,55]. However, in the fields of micro-assembly and self-calibration, how to leverage the reconfigurability of droplets to overcome the limitations of traditional rigid substrates remains a subject worthy of in-depth investigation.

Reference [56] achieved self-calibration of the position and orientation of micro-components by constructing the required droplet substrate based on the position and orientation of the manipulator’s end-effector. Guided by the position and orientation of this substrate, the restoring force of the liquid bridge drives the micro-component to rapidly align with the position and orientation of the manipulator’s end-effector, thereby achieving self-calibration of the micro-component’s position and orientation. However, this method struggles to achieve high-throughput operations. References [57,58] proposed the concept of constructing a calibration substrate based on droplet arrays and experimentally demonstrated the feasibility of the proposed self-calibration method.

Building on the above-mentioned research, this paper proposes a self-calibration method for micro-components based on reconfigurable droplet arrays. Distinct from existing work, conventional studies on controlled droplet microfluidics [59,60] primarily focus on the function of droplets as chemical reactors or screening carriers. This paper designs the droplet array itself as a shape-matched and reconfigurable calibration substrate. By regulating the spatial distribution of droplets, a liquid-bridge array matching the target micro-component is constructed, and surface tension drives the micro-component to achieve self-calibration of position and orientation. This strategy elevates droplets from “passive reaction containers” to “actively configurable functional elements”, thereby expanding the application boundary of droplet microfluidic technology in the field of micro-assembly. This paper exploits the reconfigurability of droplet arrays to investigate the self-calibration mechanism of micro-component position and orientation, establishes a mechanical model for the self-calibration process, quantitatively analyzes the mechanical coupling effect of individual droplets on the micro-component during self-calibration, and clarifies the construction method of droplet substrates. It discusses the influences of micro-component material and surface properties on self-calibration error, and validates the feasibility of the proposed method through extensive experiments. This research offers a novel perspective and theoretical foundation for developing highly adaptive and programmable micro-assembly technologies.

## 2. Self-Calibration Mechanism Based on Droplet Array

### 2.1. Structure of the Self-Calibration Platform and Generation of the Droplet Array Substrate

**Figure 1:**
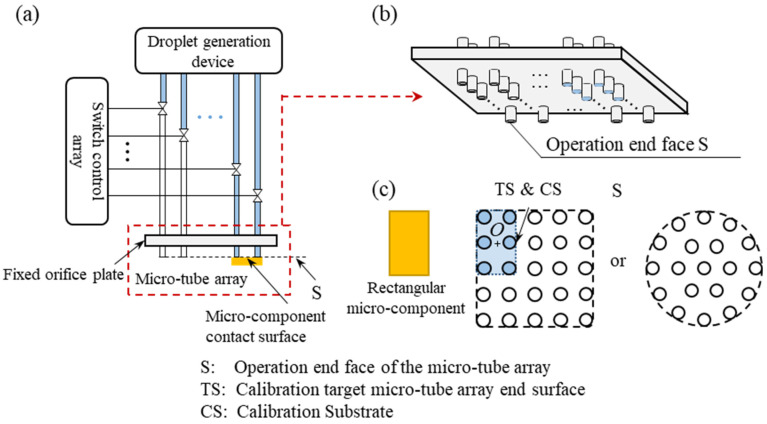
The structure of the self-calibration platform based on the droplet array. (**a**) Self-calibration platform; (**b**) Operation end face S of the micro-tube array; (**c**) Calibration target micro-tube array end surface TS and calibration substrate CS.

The self-calibration platform is composed of a micro-tube array, a droplet generation device, a switch control array, and their connecting parts, as shown in Figure 1a. The droplet generation device provides the required droplets for the micro-tube array. The switch control array can independently control the connection between the droplet generation device and each micro-tube, delivering fluid to each interconnected micro-tube and forming uniform droplets at the operation end faces of the micro-tubes. The cross-sectional dimensions of each micro-tube are consistent. Micro-tubes are uniformly embedded in a fixed orifice plate to form a micro-tube array. One end of each micro-tube in the micro-tube array is connected to the corresponding micro-tube in the droplet generation device through the switch control array, and the other end passes through the fixed orifice plate with the same protruding height. The end face of the micro-tube at this end is called the operation end face of the micro-tube array, denoted as S, as shown in Figure 1b.

According to the shape, size, position and orientation requirements of the micro-component, the corresponding micro-tubes in the micro-tube array are selected, so that the envelope shape of the end faces of the selected micro-tubes is consistent with the shape of the micro-component to be calibrated. The shape enclosed by the envelope of this operation end face constitutes the calibration target micro-tube array end surface TS. The selected micro-tube array is connected to the droplet generation device through the switch control array, forming a droplet array corresponding to the calibration target micro-tube array end surface TS. The area enclosed by the envelope of the droplet array constitutes the calibration target droplet array area, that is, the calibration substrate (CS). The shape of the calibration substrate CS is the same as that of the micro-component, and its size is slightly larger than or equal to the contact surface of the micro-component [58]. Through the above method, droplet arrays of arbitrary shapes can be constructed as the calibration substrate CS on the operation end face S of the self-calibration platform, meeting the self-calibration requirements of micro-components with different shapes and sizes. For example, if the micro-component is rectangular, a rectangular droplet array corresponding to the size of the micro-component can be selected as the calibration substrate, as shown by the blue dashed line in Figure 1c. The position and orientation of the calibration substrate CS are the target position and orientation for calibration. When the parameters such as the shape and size of the micro-component change, there is no need to change the hardware of the self-calibration platform. It is only necessary to reselect the corresponding micro-tubes to reconstruct the calibration substrate CS that meets the parameters and operation requirements of the micro-component. The arrangement mode of the micro-tube array can be changed; as shown in Figure 1c, it can be arranged into an equidistant array type or an equidistant radial type to adapt to the calibration requirements of micro-components with different shapes.

### 2.2. Self-Calibration Driving Principle

According to the target position and orientation that the micro-component needs to achieve through self-calibration, the target micro-tube array end surface TS corresponding to the shape and size of the micro-component is constructed on the self-calibration platform, and the calibration target droplet array is formed by operating the switch control array. When the micro-component comes into contact with the droplet array in the calibration substrate CS, each micro-droplet in the droplet array forms a liquid bridge in contact with the micro-component, forming a liquid bridge array. When there are asymmetric liquid bridges in the liquid bridge array, according to the principle of minimum energy, the coupled force exerted on the micro-component by the asymmetric liquid bridges drives the micro-component to change its position and orientation, so that the position and orientation of the micro-component are consistent with those of the calibration substrate CS, realizing the self-calibration of the position and orientation between the micro-component and CS.

### 2.3. Self-Calibration Process

#### 2.3.1. The Force Exerted by the Liquid Bridge on the Micro-Component

**Figure 2:**
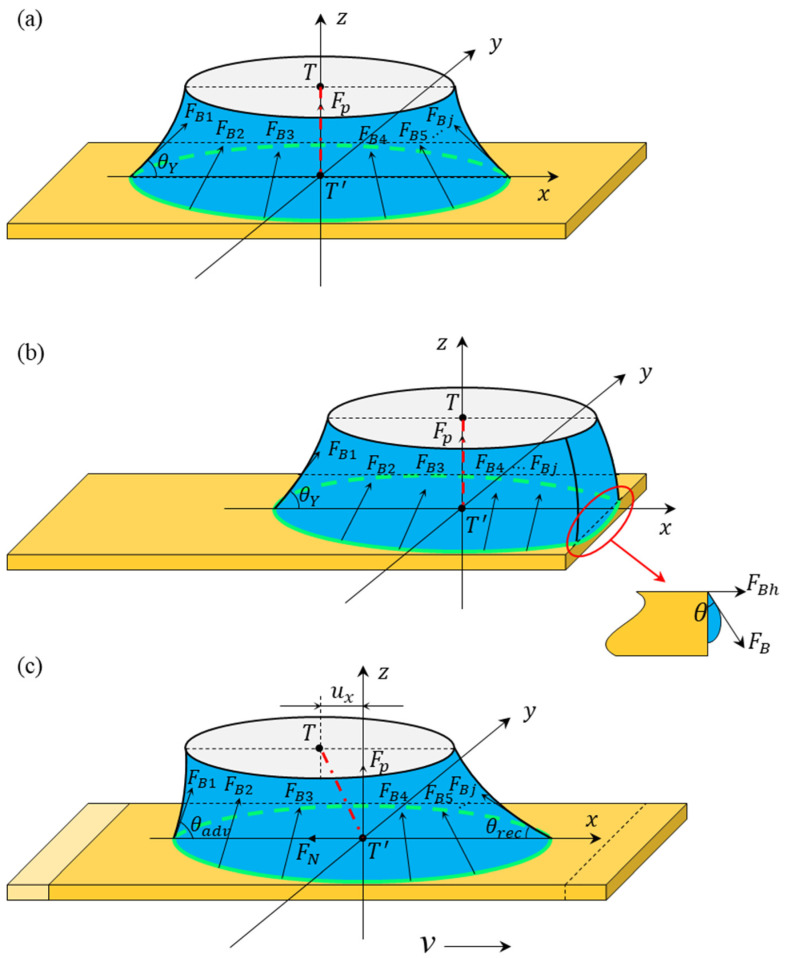
Force exerted by the liquid bridge on the micro-component (The gray region represents the substrate, the blue region denotes liquid bridges, the yellow region indicates the micro-component. The red dashed line indicates the centerline of the liquid bridge, the bright green curve indicates the three-phase contact line on the micro-component surface. $\theta_{Y}$ is the Young contact angle, and $\theta_{adv}$, $\theta_{rec}$ are the advancing and receding contact angles). (**a**) Symmetric case; (**b**) asymmetric case due to edge wetting; (**c**) inclined case with adhesion force. See text for detailed definitions of symbols.

The morphology of the resulting liquid bridge varies depending on the droplet’s position on the contact surface of the micro-component, which in turn dictates the forces it exerts. Figure 2 illustrates the forces exerted on the micro-components by the liquid bridges. $F_{p}$ denotes the force induced by the internal negative pressure of the liquid bridge, acting on the wetted area of the micro-component along the *z*-axis of the liquid bridge. $F_{Bj}$ represents the surface tension acting along the three-phase contact line on the upper surface of the micro-component, directed tangentially to the liquid–gas interface. The resultant surface tension force $F_{B}=\sum F_{Bj}$, $F_{B}$ can be decomposed into its Cartesian components $\left(F_{Bx},F_{By},F_{Bz}\right)$. The center of the upper liquid–solid interface of the liquid bridge (i.e., the end face of the micro-tube) is denoted as T, and the center of the lower liquid–solid interface, which corresponds to the completely wetted area of the liquid bridge on the micro-component, is denoted as T’. The line connecting T and T’ is referred to as the centerline of the liquid bridge. When relative motion occurs between the upper and lower solid surfaces, meaning the centerline $TT^{\prime }$ is no longer perpendicular to the micro-component surface, the liquid bridge exerts an adhesion force $F_{N}$ on the micro-component. Consequently, the general form of the force equation acting on the micro-component is expressed as:

(1) $$\begin{array}{c} \left\{\begin{array}{c} F_{x}=F_{Bx}+F_{Nx}\\ F_{y}=F_{By}+F_{Ny}\\ F_{z}=F_{Bz}+F_{p} \end{array}\right. \end{array}$$

As the droplet spreads freely across the micro-component, its three-phase contact line forms a circle, yielding a symmetric liquid bridge. When the centerline $TT^{\prime }$ is strictly perpendicular to the component surface, as depicted in Figure 2a, the horizontal components of the surface tension $F_{B}$ within the xy-plane completely cancel each other out $\sum F_{Bj}=0$. Under these conditions, the net force exerted by the liquid bridge on the micro-component is given by

(2) $$\begin{array}{c} \left\{\begin{array}{c} F_{x}=0\\ F_{y}=0\\ F_{z}=F_{Bz}+F_{p} \end{array}\right. \end{array}$$

As the droplet spreads across the micro-component surface, if it reaches the micro-component edge or encounters geometric boundary constraints, as illustrated in Figure 2b, an asymmetric liquid bridge emerges. When the liquid bridge wets the lateral surface of the micro-component, the total exerted force must account for this lateral wetting effect. Specifically, the horizontal force induced by lateral wetting is given by $F_{Bh}=\gamma l_{c}\sin\theta$, indicating that its magnitude is proportional to the liquid surface tension γ, the lateral wetted perimeter $l_{c}$, and the sine of the contact angle θ. The three-phase contact line on the upper surface of the micro-component forms a circular segment. Both the geometric asymmetry of this contact line and the variation in the contact angle on the lateral surface result in a non-zero resultant surface-tension force $F_{B}$. Owing to the edge effect of the liquid bridge, the horizontal force exerted on the micro-component by the liquid bridge is $F_{h}=F_{Bx}+F_{Bh}=F_{Bx}+\gamma l_{c}\sin\theta$. Assuming the micro-component operates as a thin sheet with an extremely small thickness, its lateral wetted perimeter $l_{c}$ is negligible compared to the wetted perimeter on the upper surface. Consequently, the horizontal force contributed by lateral wetting can be disregarded relative to the horizontal force acting on the upper surface. Under this condition, the net force exerted by the liquid bridge on the micro-component is

(3) $$\begin{array}{c} \left\{\begin{array}{c} F_{x}=F_{Bx}\\ F_{y}=0\\ F_{z}=F_{Bz}+F_{p} \end{array}\right. \end{array}$$

If relative motion occurs between the upper and lower solid surfaces of the liquid bridge, the pinning effect causes the central line $TT^{\prime }$ of the liquid bridge to be non-perpendicular to the micro-component surface. When the upper and lower solid surfaces move relatively along the x-direction, a deviation $u_{x}$ exists between the liquid bridge central line and the *z*-axis, corresponding to the liquid bridge state shown in Figure 2c. Consequently, the liquid–solid interface of the liquid bridge will generate a horizontal adhesion force $F_{N}$ on the micro-component. This force acts on the contact plane of the micro-component. Its magnitude is related to the material of the micro-component surface (Young’s contact angle) and its roughness (advancing and receding contact angles), and its direction is opposite to the movement tendency of the micro-component. Meanwhile, due to the variation in the contact angle on the lateral surface of the liquid bridge, the resultant surface tension force acting on the three-phase contact line is $F_{B}\neq 0$, and its direction is opposite to the movement trend of the micro-component. At this moment, the force exerted by the liquid bridge on the micro-component is

(4) $$\begin{array}{c} \left\{\begin{array}{c} F_{x}=F_{Bx}+F_{Nx}\\ F_{y}=0\\ F_{z}=F_{Bz}+F_{p} \end{array}\right. \end{array}$$

**Figure 3:**
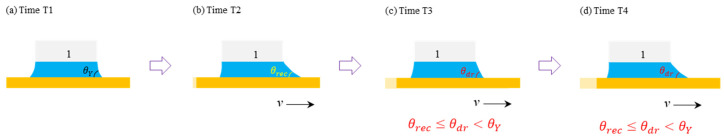
Schematic of Liquid-Bridge Sliding.

Under the ideal condition of a perfectly smooth micro-component surface, $F_{N}\approx 0$. The liquid bridge maintains a symmetric configuration as its three-phase contact line moves across the micro-component surface, with its central axis $TT^{\prime }$ consistently perpendicular to the micro-component surface. In practice, however, real micro-component surfaces possess inherent roughness, giving rise to advancing and receding contact angles for liquid bridges. The resultant pinning effect hinders the motion of the three-phase contact line on the micro-component surface, leading to variations in liquid-bridge morphology and the dynamic contact angle of the liquid bridge at the micro-component surface, as illustrated in Figure 3. When the contact angle decreases to the receding contact angle $\theta_{rec}$, the three-phase contact line slides along the micro-component surface. Following sliding, the dynamic receding contact angle $\theta_{dr}$ of the liquid bridge varies within the range $\theta_{rec}\leq \theta_{dr}<\theta_{Y}$, as shown in Figure 3c. As the micro-component continues to move, the three-phase contact line becomes pinned again, and the dynamic receding contact angle once more decreases. This process repeats cyclically, whereby the dynamic receding contact angle decreases until reaching its limiting receding value, followed by sliding of the three-phase contact line and a subsequent increase in the dynamic receding contact angle.

Since deviations in the vertical (z-axis) direction are largely eliminated once the micro-component contacts the droplets, self-calibration mainly targets translational and orientational adjustments in the horizontal plane. Accordingly, subsequent analysis focuses on the horizontal components of surface tension within the xy-plane as well as horizontal adhesion forces.

#### 2.3.2. Analysis of the Self-Calibration Process

**Figure 4:**
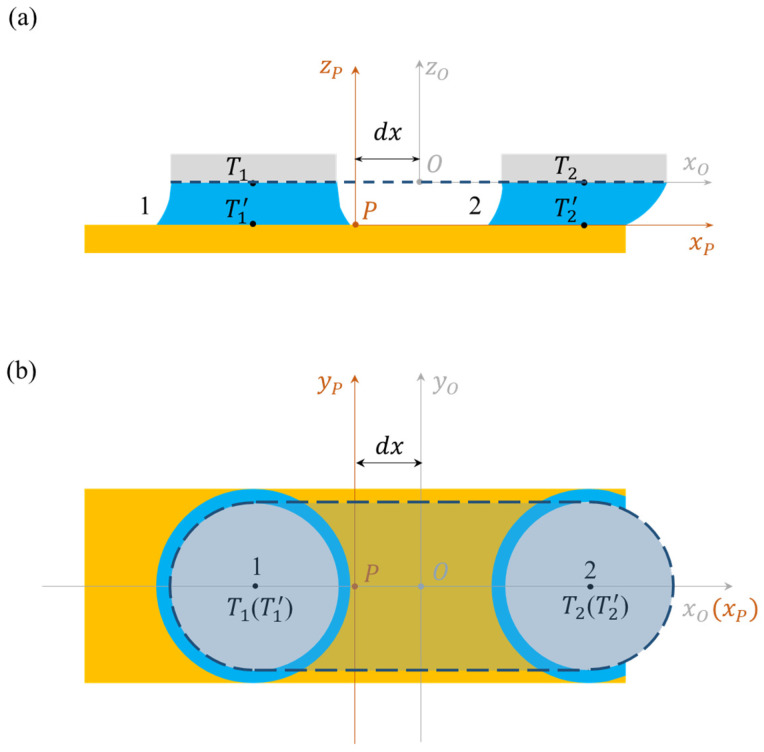
Establishment of Coordinate Systems for the Self-Calibration System (The gray area denotes the selected micro-tube array TS, the blue area represents the liquid-bridge array, dashed lines represent the calibration substrate CS, and the yellow area represents the micro-component). (**a**) Front view; (**b**) Top view.

Taking a two-droplet substrate as an example, the self-calibration process of thin-sheet micro-components is illustrated. The coordinate system of the self-calibration system is shown in Figure 4. A coordinate system $\Sigma O\left(x_{O},y_{O},z_{O}\right)$ is established with the geometric center O of the calibration substrate CS as the origin, and another coordinate system $\Sigma P\left(x_{P},y_{P},z_{P}\right)$ for the micro-component contact surface is defined with the geometric center P of the micro-component upper contact surface as the origin. Deviations between the micro-component contact surface and the calibration substrate CS are represented by positional and orientational deviations between the two coordinate systems ΣO and ΣP. Here, $R_{1}$ denotes the upper liquid–solid interface radius of the liquid bridge, and $R_{2}$ is the free spreading radius of the liquid bridge on the micro-component surface. For thin-sheet micro-components, attention is mainly focused on deviations within the xy-plane of the micro-component.

Assume that the micro-component has an initial positional deviation dx from the calibration substrate CS along the x-direction. Two liquid bridges, denoted as Liquid Bridge 1 and Liquid Bridge 2, form after contact between the micro-component and the calibration substrate CS, as shown in Figure 4a. Owing to the initial deviation dx, the three-phase contact line of Liquid Bridge 1 spreads into a circle on the micro-component surface, whereas that of Liquid Bridge 2 spreads into a circular segment, as illustrated in Figure 4b.

The initial positional and orientational deviations of the micro-component in the xy-plane are expressed as $\left(dx,dy,d\theta \right)$. The residual errors between the micro-component and the calibration substrate CS after self-calibration are defined as self-calibration errors $\left(\delta x,\delta y,\delta \theta \right)$, where δx and δy represent positional errors, and δθ denotes the orientational error of the micro-component.

According to the analysis in Section 2.3.1, the resultant forces exerted by the two liquid-bridge arrays on the micro-component along each axis are

(5) $$\begin{array}{c} \left\{\begin{array}{c} F_{x}=F_{x1}+F_{x2}\\ F_{y}=F_{y2}+F_{y2}\\ F_{z}=F_{z1}+F_{z2} \end{array}\right. \end{array}$$

**Figure 5:**
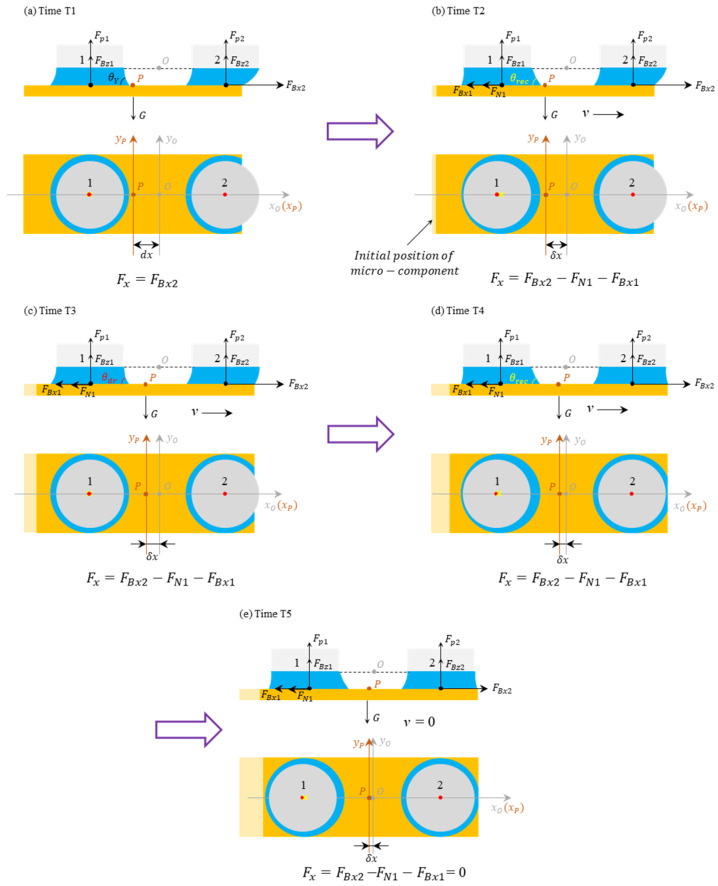
Schematic diagram of the self-calibration process for the position deviation of the rectangular micro-component (The light yellow shape represents the initial position of the micro-component, the red dot indicates the center of the upper liquid–solid interface of the liquid bridge, the yellow ‘×’ mark indicates the center of the lower liquid–solid interface of Liquid Bridge 1).

During the self-calibration process, as the morphology of the two liquid bridges changes, the forces exerted by the liquid bridges on the micro-component also vary. Assuming an initial deviation dx in the x-direction between the initial position of the micro-component and the calibration substrate CS, as shown in Figure 5a, at time T1 (initial state), Liquid Bridge 1 is fully spread on the micro-component, with a circular three-phase contact line and its centerline perpendicular to the micro-component. At this point, Liquid Bridge 1 is symmetric, and the components of surface tension $F_{B}$ in the x and y directions are $F_{Bx1}=F_{By1}=0$. The adhesion force in the x-direction $F_{Nx1}=0$, and the resultant forces exerted by Liquid Bridge 1 on the upper surface of the micro-component in the x and y directions are $F_{x1}=0,F_{y1}=0$. Liquid Bridge 2 spreads on the micro-component into an approximately truncated circular shape, with an asymmetric three-phase contact line, forming an asymmetric liquid bridge. The resultant surface tension force of Liquid Bridge 2 on the upper surface of the micro-component in the x-direction is $F_{Bx2}\neq 0$, while that along the y-direction is $F_{By2}=0$. Meanwhile, the resultant force in the z-direction from Liquid Bridges 1 and 2($F_{z1}$, $F_{z2}$), balance the gravitational force of the micro-component. Thus, the resultant force exerted by Liquid Bridges 1 and 2 on the micro-component at time T1 is obtained as

(6) $$\begin{array}{c} \left\{\begin{array}{c} F_{x}=F_{Bx2}\\ F_{y}=0\\ F_{z}=F_{Bz1}+F_{Bz2}+F_{p1}+F_{p2}=G \end{array}\right. \end{array}$$

Driven by the resultant force of Liquid Bridges 1 and 2, the micro-component is rapidly adsorbed and starts to move along the x-axis under $F_{x}$.

As the micro-component moves, dx decreases and the asymmetry of Liquid Bridge 2 weakens. Under ideal conditions, the surface of the micro-component is smooth, with no influence from surface roughness. At this point, the three-phase contact line of Liquid Bridge 1 will slide on the surface of the micro-component, and its centerline remains always perpendicular to the micro-component surface. That is, Liquid Bridge 1 remains a symmetric liquid bridge throughout the calibration process. Under the restoring force of Liquid Bridge 2, the position and orientation of the micro-component automatically calibrate with the calibration substrate CS.

However, the actual surface of a micro-component is not absolutely smooth; it exhibits an advancing contact angle and a receding contact angle. The centerline of Liquid Bridge 1 tilts as the micro-component moves, causing Liquid Bridge 1’s morphology to change. Liquid Bridge 1 transitions from a symmetric to an asymmetric liquid bridge, generating an adhesion resistance $F_{N1}$ that impedes motion, in a direction opposite to the restoring force generated by Liquid Bridge 2. Simultaneously, after the tilt of Liquid Bridge 1, the surface tension acting on the lateral surface of the liquid bridge at the three-phase contact line also becomes asymmetric. The resultant horizontal force component in the xy-plane from the lateral surface tension of Liquid Bridge 1 also points in the direction opposite to the restoring force of Liquid Bridge 2. At this moment, the resultant force in the x-direction acting on the micro-component from Liquid Bridges 1 and 2 is $F_{x}=F_{Bx2}{-F}_{N1}-F_{Bx1}$. When $F_{x}>0$, the micro-component continues to move in the x-direction under the action of $F_{x}$.

As the micro-component continues to move, the tilt of the liquid bridge centerline will gradually increase. When the right-side contact angle of Liquid Bridge 1 decreases to the receding contact angle $\theta_{rec}$, corresponding to the situation at time T2 shown in Figure 5b, if the resultant force $F_{x}$ acting on the micro-component is still greater than zero at this moment, the three-phase contact line of Liquid Bridge 1 will slide on the contact surface of the micro-component, and the contact angle on the right side increases to an angle between $\theta_{rec}$ and $\theta_{Y}$, as shown in Figure 5c. As the self-calibration progresses, whenever Liquid Bridge 1 reaches the receding contact angle again, the three-phase contact line slides. The three-phase contact line periodically repeats the process of slide-stick-slide on the micro-component surface, as depicted in Figure 5d. This continues until the restoring force generated by Liquid Bridge 2 equals the resistance generated by Liquid Bridge 1, i.e., $F_{x}=F_{Bx2}{-F}_{N1}-F_{Bx1}=0$, at which point the liquid bridge system reaches equilibrium, as shown in Figure 5e, and the calibration is complete. At this moment, the self-calibration position error is denoted as $\left(\delta x,\delta y\right)$.

**Figure 6:**
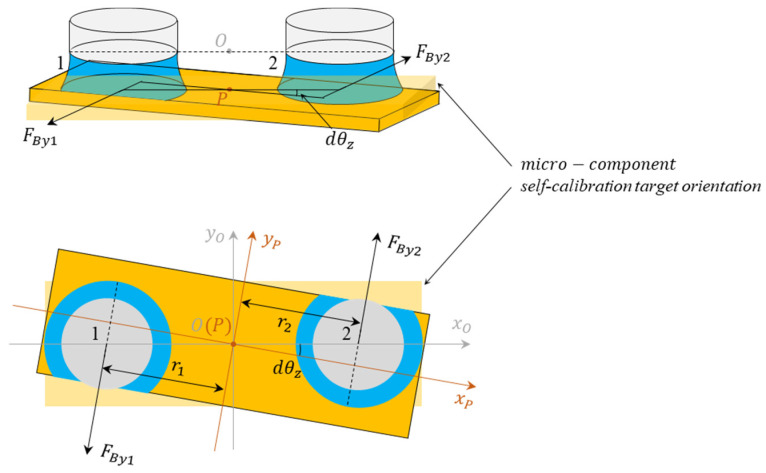
Schematic of forces exerted during the rotational self-calibration of a rectangular micro-component.

When there is an orientation deviation dθ between the micro-component and the calibration substrate CS, the forces exerted on the micro-component by Liquid Bridges 1 and 2 are shown in Figure 6. $F_{By1}$ and $F_{By2}$ represent the forces acting on the micro-component by Liquid Bridges 1 and 2 in the xy-plane. $F_{By1}$ and $F_{By2}$ form a couple, driving the micro-component to rotate counterclockwise and quickly calibrate with the orientation of the calibration substrate CS to reach a new equilibrium. The orientation error after self-calibration is denoted as δθ.

## 3. Force Solution of Micro-Components Based on Energy Method

According to the analysis in Section 2, during the self-calibration process with position deviation, the asymmetric liquid bridge force of Liquid Bridge 2 drives the micro-component to calibrate toward the calibration substrate CS, which is defined as the restoring force, while Liquid Bridge 1 changes from a symmetric liquid bridge to an asymmetric one during the self-calibration of the micro-component, and its effect of impeding the self-calibration of the micro-component is defined as the resistance.

### 3.1. Mathematical Description of Forces and Moments Acting on Micro-Components

Assume that there exists a relative position deviation $\left(dx,dy\right)$ and an orientation deviation dθ between the micro-component and the calibration substrate CS. According to the conservation of energy, the work done by the restoring force (moment) over this displacement equals the variation in the liquid bridge free energy E. The restoring force ${\left(F_{x},F_{y}\right)}^{T}$ generated by each liquid bridge on the contact surface of the micro-component are expressed as

(7) $$\begin{array}{c} \left\{\begin{array}{c} F_{xi}=\frac{\partial E_{i}}{\partial x}\\ F_{yi}=\frac{\partial E_{i}}{\partial y} \end{array}\right. \end{array}$$

and restoring moment T is expressed as

(8) $$\begin{array}{c} T_{i}=\frac{\partial E_{i}}{\partial \theta } \end{array}$$

where $E_{i}$ denotes the free energy of the i-th liquid bridge, which consists of two parts: the free energies of liquid–solid, liquid–vapor and vapor–solid interfaces, and the volume free energy of liquid bridge i. It can be explicitly expressed as

(9) $$E_{i}=\sum_{g=1}^{n}\gamma_{g}A_{gi}-\sum_{h=1}^{m}p_{hi}V_{hi}=\gamma_{lst}A_{lst}+\gamma_{vst}A_{vst}+\gamma_{lsb}A_{lsb}+\gamma_{vsb}A_{vsb}+\gamma_{lv}A_{lv}-p_{l}V_{l}-p_{v}V_{v}$$

where g denotes each interface of the liquid bridge, h represents the liquid–vapor two-phase state, $A_{lst}$ is the upper liquid–solid interfacial area of the liquid bridge, and $\gamma_{lst}$ is the corresponding interfacial tension; $A_{vst}$ is the interfacial area between ambient gas and the end face of the micro-tube array, and $\gamma_{vst}$ is the corresponding interfacial tension; $A_{lsb}$ is the lower liquid–solid interfacial area of the liquid bridge, and $\gamma_{lsb}$ is the corresponding interfacial tension; $A_{vsb}$ is the interfacial area between the ambient gas and the upper surface of the micro-component, and $\gamma_{vsb}$ is the corresponding interfacial tension; $A_{lv}$ is the contact surface area between the liquid in the liquid bridge and the ambient gas, and $\gamma_{lv}$ is the corresponding liquid–vapor surface tension; $V_{l}$ is the volume of the liquid bridge, $p_{l}$ is the internal pressure of the liquid, $V_{v}$ is the gas volume within the liquid-bridge system, and $p_{v}$ is the gas pressure.

Droplets on the substrate are generated by micro-tubes with identical parameters, such that the upper liquid–solid interfacial area $A_{lst}$ of each liquid bridge, i.e., the end-face area of the micro-tube remains constant. The operating end face of each micro-tube is not exposed to gas, so $A_{vst}=0$. The interfacial tension $\gamma_{vsb}$ between the micro-component surface and the ambient gas is extremely small and generally neglected. Controlled by the droplet generation device, the liquid-bridge volume $V_{l}$ used for calibration and the gas volume $V_{v}$ within the system are both constant. Accordingly, Equation (9) can be simplified after differentiation as

(10) $$\begin{array}{c} \delta E_{i}=\gamma_{ls}\delta A_{lsbi}+\gamma_{lv}{\delta A}_{lvi} \end{array}$$

In subsequent calculations, only the variations in the lower liquid–solid interfacial area $\delta A_{lsb}$ (hereinafter abbreviated as $\delta A_{ls}$) and the lateral liquid–vapor surface area ${\delta A}_{lv}$ of the liquid bridge need to be determined to obtain the corresponding free-energy variation in the liquid-bridge system. Substituting these into Equations (7) and (8) yields the restoring force and restoring torque of the liquid bridge.

When the micro-component only exhibits an orientational deviation about the z-axis relative to the calibration substrate CS, as shown in Figure 6, Liquid Bridges 1 and 2 form a couple. Thus, only the values of $A_{ls}$ and $A_{lv}$ for one liquid bridge need to be calculated. The surface free-energy variation δE of the liquid-bridge array can be expressed as

(11) $$\begin{array}{c} \delta E=\delta E_{1}+\delta E_{2}=2(\gamma_{ls}\delta A_{ls1}+\gamma_{lv}{\delta A}_{lv1}) \end{array}$$

Therefore, the resultant restoring torque acting on the micro-component is

(12) $$\begin{array}{c} T=\frac{\partial E}{\partial \theta }=2(\gamma_{ls}\frac{\partial A_{ls1}}{\partial \theta }+\gamma_{lv}\frac{{\partial A}_{lv1}}{\partial \theta }) \end{array}$$

### 3.2. Calculation of the Liquid Bridge Restoring Force

Consider the liquid bridge system illustrated in Figure 4, where two liquid bridges form between the micro-component and the calibration substrate CS. When the micro-component deviates within the calibratable range along the x-direction [58], Liquid Bridge 2 generates a self-calibrating restoring force. Based on geometric relationships, the lower liquid–solid interfacial area $A_{ls2}$ and lateral liquid–vapor surface area $A_{lv2}$ of Liquid Bridge 2 can be derived. Using the values of $A_{ls2}$ and $A_{lv2}$ obtained at different moments, the free-energy variation in Liquid Bridge 2 is acquired by substituting into Equation (10), and the restoring force of Liquid Bridge 2 is further calculated via substitution into Equation (7).

(13) $$\begin{array}{c} F_{x2}=-2\sqrt{2R_{2}}\left(\gamma_{ls}+\gamma_{lv}\right)\sqrt{\delta x}+\frac{\sqrt{2}}{2}R_{2}^{-1/2}\left(\gamma_{ls}+\gamma_{lv}\right)\delta x^{3/2} \end{array}$$

where $R_{2}$ is the free spreading radius of the liquid bridge on the micro-component, which can be calculated according to the liquid bridge volume V, the upper liquid–solid interface radius $R_{1}$ of the liquid bridge and the contact angle $\theta_{Y}$.

The calculation method of the restoring force when the micro-component has an initial deviation in the y-direction is the same as that for the x-direction described above.

### 3.3. Solution of Resistance Force Exerted by Liquid Bridge on Micro-Component

When the centerline of the liquid bridge is inclined, the liquid bridge exerts a horizontal adhesive force $F_{N}$ on the micro-component. Taking the liquid bridge system shown in Figure 4 as an example, as the restoring force of Liquid Bridge 2 drives the micro-component to move, Liquid Bridge 1 starts to incline. When the contact angle on the right side of Liquid Bridge 1 reaches the receding contact angle, the static resistance reaches the maximum, and Liquid Bridge 1 will slide on the surface of the micro-component.

Liquid Bridge 1 is simplified as an oblique conical frustum model [61] for calculation. According to the geometric relationship, the lateral surface area $A_{lv1}$ of Liquid Bridge 1 can be obtained. During the calibration process, the contact area $A_{ls1}$ between Liquid Bridge 1 and the micro-component remains constant due to pinning effects. According to Equation (10), the surface free-energy variation in Liquid Bridge 1 can be expressed as

(14) $$\begin{array}{c} {\delta E}_{1}=\gamma_{lv}{\delta A}_{lv1} \end{array}$$

Substituting Equation (14) into Equation (7) yields the horizontal resistance force exerted by Liquid Bridge 1 on the micro-component

(15) $$\begin{array}{c} F_{x1}=-\left(\frac{\gamma_{lv}\pi }{H^{2}{\left(1+\xi^{2}\right)}^{\frac{3}{2}}}\int_{0}^{H}\left[R_{2}-z\frac{\left(R_{2}-R_{1}\right)}{H}\right]dz\right)\cdot u_{x} \end{array}$$

where H is the height of the liquid bridge, $\xi =\left(R_{2}-R_{1}\right)/H$, and $u_{x}$ is the projection of the centerline $T_{1}T_{1}^{\prime }$ of Liquid Bridge 1 along the x-direction on the xy-plane.

## 4. Analysis and Discussion of Self-Calibration Errors

### 4.1. Generation and Evaluation of Self-Calibration Error

According to the analysis in Section 2.3.2 and the solution in Section 3, the calibration motion of the micro-component in the xy-plane results from the combined forces exerted on it by each liquid bridge in the liquid bridge array.

In the two-liquid-bridge model shown in Figure 4, self-calibration can be realized within the pinning range of Liquid Bridge 1 if the initial positional deviation of the micro-component in the negative x-direction is less than the maximum allowable inclined offset distance $u_{xmax}$ of the resistance liquid bridge. During calibration, as the micro-component moves along the x-direction, the resistance force of Liquid Bridge 1 increases linearly and acts opposite to the restoring force of Liquid Bridge 2. When the resistance force of Liquid Bridge 1 is equal in magnitude and opposite in direction to the restoring force of Liquid Bridge 2, the liquid-bridge system reaches equilibrium and self-calibration terminates. At this moment, the positional error between the micro-component and the target calibration position is defined as the self-calibration positional error induced by the resistance within the pinning range of Liquid Bridge 1.

**Figure 7:**
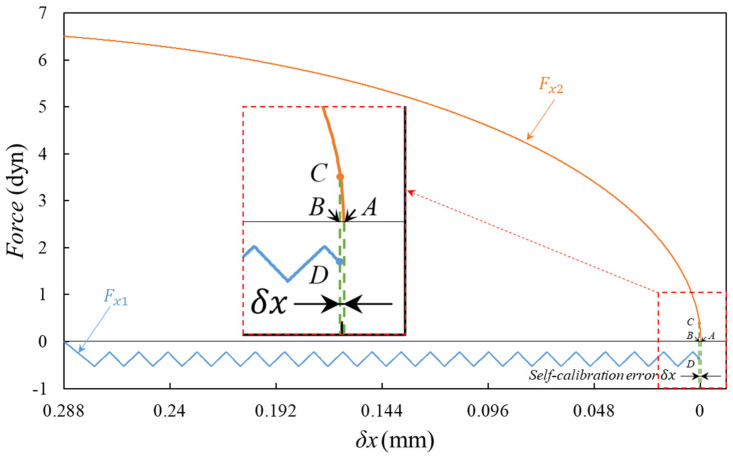
Variations of ${F_{x1},F}_{x2}$ during the self-calibration process of the micro-component in the x-direction (Liquid bridge volume V=32.72 nL, micro-tube radius $R_{1}=0.25\;mm$, Young’s contact angle $\theta_{Y}=75{}^{\circ}$, receding contact angle $\theta_{rec}=70{}^{\circ}$, $\gamma_{lv}=72.75\;mN/m$,$\gamma_{ls}=33.68\;mN/m$).

If the micro-component is offset in the negative x-direction to the maximum calibratable position of the dual-liquid-bridge calibration substrate [58], i.e., the midline position of Liquid Bridge 2, the self-calibrating restoring force $F_{x2}$ of the micro-component and the liquid-bridge resistance force $F_{x1}$ can be calculated using Equations (13) and (15). Under ideal conditions, the surface of the micro-component is perfectly smooth, and the resistance force of Liquid Bridge 1 on the contact surface of the micro-component can be neglected ($F_{x1}=0$). The micro-component will rapidly calibrate to the target position and orientation under the restoring force $F_{x2}$ of Liquid Bridge 2, with a self-calibration error of zero, as shown at Point A in Figure 7. Considering the influence of micro-component surface roughness, the resistance exerted by Liquid Bridge 1 on the micro-component varies periodically as Liquid Bridge 1 slides along the micro-component surface, and acts in the opposite direction to the restoring force of Liquid Bridge 2. When the resistance force of Liquid Bridge 1 (Point D) and the restoring force of Liquid Bridge 2 (Point C) are equal in magnitude and opposite in direction, the resultant force acting on the micro-component becomes zero at Point B. The liquid bridge system reaches a new equilibrium and the self-calibration ceases. The corresponding error δx is defined as the theoretical error of self-calibration.

### 4.2. Influencing Factors on Self-Calibration Errors

#### 4.2.1. Influence of Micro-Component Material on Liquid Bridge Resistance

**Figure 8:**
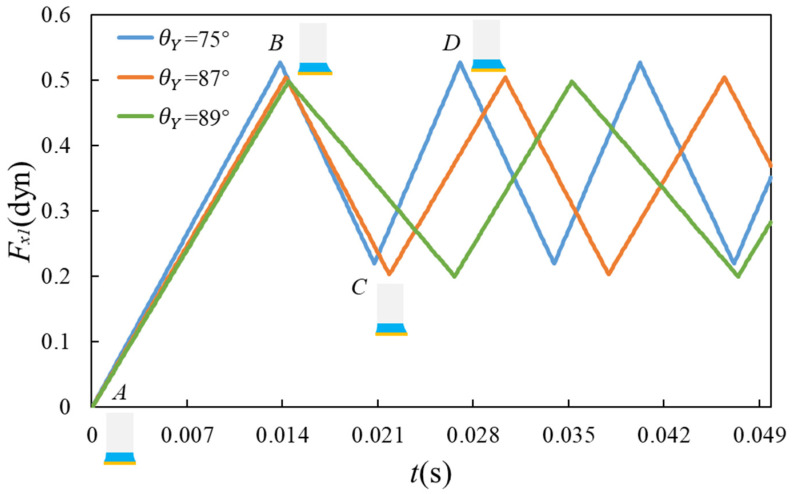
Periodic variation in resistance $F_{x1}$ of Liquid Bridge 1 on micro-components with different materials (Calculated parameters of Liquid Bridge 1: $V=32.72\;nL,\gamma_{lv}=72.75\;mN/m,R_{1}=0.25\;mm$, surface roughness of the micro-components is identical).

When the parameters of the micro-tube array and the fluid are fixed, the parameters of the micro-component directly affect the resistance exerted by the liquid bridge on it. When the material of the micro-component is different, the contact angle of the liquid bridge on the surface of the micro-component varies accordingly. With the same droplet volume, as the contact angle of the liquid bridge on the micro-component surface increases, the spreading radius of the liquid bridge decreases, and its height increases. According to Equation (15), the periodic variation in the resistance $F_{x1}$ exerted by Liquid Bridge 1 on micro-components of three different materials (with contact angles of 75°, 87°, and 89°) during sliding can be obtained, as shown in Figure 8.

In Figure 8, the blue curve represents the resistance curve of Liquid Bridge 1 when the contact angle on the micro-component is $\theta_{Y}=75{}^{\circ}$. In the initial state, Liquid Bridge 1 is symmetric, with $F_{x1}=0$ (point A). Under the restoring force of Liquid Bridge 2, the micro-component moves in the x-direction. The three-phase contact line of Liquid Bridge 1 on the surface of the micro-component remains unchanged, but as the micro-component moves, Liquid Bridge 1 tilts, causing the liquid–gas interface to become asymmetric. This generates a resistance force $F_{x1}$ that hinders the motion of the micro-component, and the resistance value gradually increases. According to Equation (15), when the right-side contact angle of Liquid Bridge 1 decreases to the receding contact angle due to tilting, $F_{x1}$ reaches its maximum value (point B). As the micro-component continues to move, the three-phase contact line of Liquid Bridge 1 begins to slide on the micro-component, causing the right-side contact angle to gradually increase to the dynamic receding contact angle. Consequently, $F_{x1}$ gradually decreases to point C. As the self-calibration motion continues, the right-side contact angle of Liquid Bridge 1 decreases again to the receding contact angle, and the above process repeats, forming a periodic variation.

The contact angles of the liquid bridge on the micro-components are different, and the period of resistance variation in Liquid Bridge 1 is also different. As the Young’s contact angle $\theta_{Y}$ increases, the adhesion effect of the liquid bridge on the micro-component surface weakens, resulting in a decreasing trend in the peak resistance and a corresponding lengthening of the period. Different materials of the micro-component lead to different resistance forces generated by the liquid bridge. Different materials of the micro-components lead to different resistances generated by the liquid bridge. For a given centerline offset $u_{x}$ of the liquid bridge, the resistance acting on the micro-component decreases as the contact angle of the material increases.

#### 4.2.2. Influence of Micro-Component Material on Liquid Bridge Restoring Force

**Figure 9:**
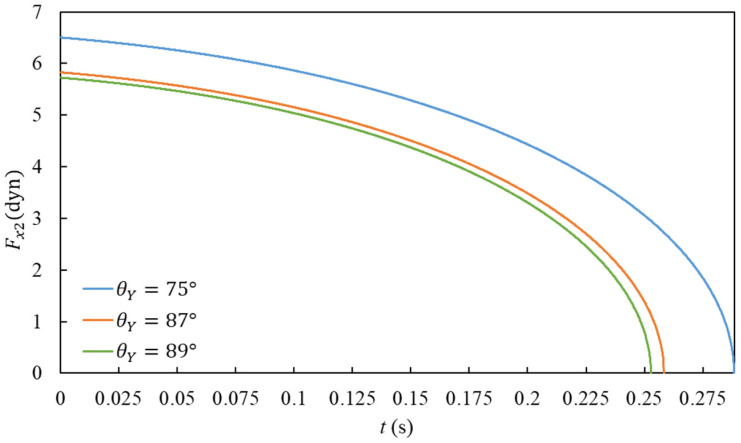
Restoring force $F_{x2}$ of Liquid Bridge 2 on different materials (Calculated parameters of Liquid Bridge 2: V=32.72 nL, $\gamma_{lv}=72.75\;\mathrm{mN}/m$).

As shown in Figure 9, the time-dependent restoring force $F_{x2}$ calculated by Equation (13) is presented for micro-components made of three different materials with contact angles of 75°, 87°, and 89°, respectively. In the initial state, when the micro-component is at the maximum positional deviation dx along the x-direction, the restoring force $F_{x2}$ reaches its maximum value. As the micro-component moves during calibration, $F_{x2}$ gradually decreases until $F_{x2}=0$. A larger Young contact angle $\theta_{Y}$ of the material corresponds to a smaller restoring force $F_{x2}$ and a narrower calibratable range. This demonstrates that better hydrophilicity of the micro-component facilitates self-calibration.

#### 4.2.3. Influence of Micro-Component Surface Roughness on Liquid Bridge Resistance

**Figure 10:**
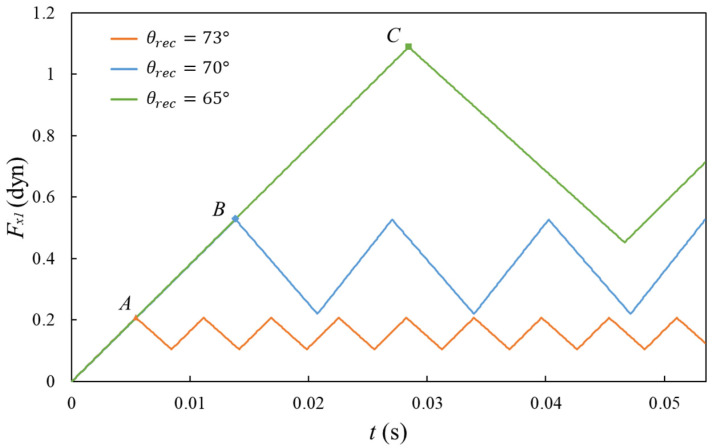
Resistance force $F_{x1}$ of Liquid Bridge 1 on surfaces with different roughness.

When the material of the micro-component is the same but the surface roughness changes, the advancing and receding contact angles vary [62,63,64], inducing morphological variations in the liquid bridge. The corresponding resistance change in Liquid Bridge 1 can be calculated using Equation (15). For simplicity and without loss of generality, it is assumed that when $\theta_{Y}=75{}^{\circ}$, the three receding contact angles $\theta_{rec}$ corresponding to increasing roughness are 73°, 70°, and 65°, respectively. The calculated curves are shown in Figure 10. The greater the roughness of the material, the smaller the corresponding receding contact angle, and the larger the maximum resistance force of the liquid bridge. Different roughness levels lead to different critical sliding positions of Liquid Bridge 1 on the micro-component surface, as indicated by points A, B, and C in the figure. The orange curve corresponds to lower roughness, with a larger receding contact angle and smaller ultimate resistance. In this case, the liquid bridge easily slides on the micro-component surface, creating a cyclical sawtooth-like fluctuation in the curve. The rougher the surface, the longer the stick-slide period of the liquid bridge.

Since Liquid Bridge 2 does not slip during the calibration process, the surface roughness of the micro-component has no effect on the restoring force of Liquid Bridge 2.

It can be seen from the above analysis that the theoretical self-calibration error is related to the micro-component material (contact angle), surface roughness (receding contact angle), and initial deviation magnitude.

## 5. Experimental Research

### 5.1. Experimental Principle and Device

**Figure 11:**
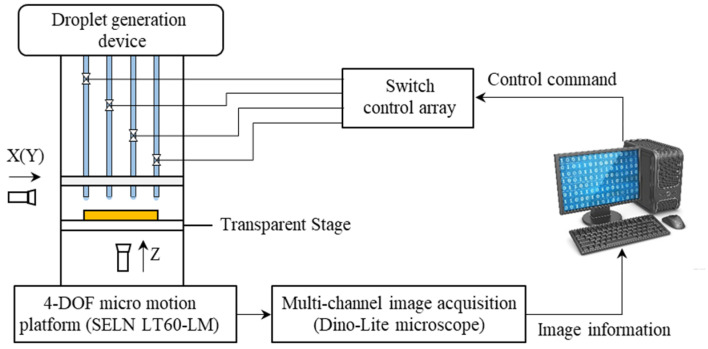
Schematic Diagram of the Experiment.

The experiment adopts a 4 × 4 micro-tube array. Each micro-tube in the array has an identical cross-sectional dimension, and its end face is polished to ensure flatness. The outer diameter of each micro-tube is 0.5 mm, the inner diameter is 0.3 mm, and the center-to-center distance between adjacent micro-tubes is 1 mm. Droplets in each micro-tube can be individually controlled by switches. Distilled water is used as the experimental liquid, and the droplet volume at the end face of each micro-tube is generally 0.03 μL. All experiments were carried out under constant temperature and humidity conditions with a controlled temperature of 20 °C and a relative humidity of 45%. Considering the losses such as the evaporation of the liquid during the experiment, the switch is reopened and closed before each experiment to replenish the liquid to keep the volume of the liquid droplets basically unchanged during the experiment. The micro-component is placed on a transparent stage, whose position is adjusted by a precision motion stage with a travel range of 3 mm and a positioning accuracy of 0.01 mm. The experimental process is observed via three-channel Dino-Lite (AM8917MZT) microscopes(AnMo Electronics Corporation, New Taipei City, Taiwan, China), with images displayed and captured on a computer to facilitate real-time observation and measurement by operators. The experimental principle is illustrated in Figure 11.

### 5.2. Position and Orientation Self-Calibration Experiment

**Figure 12:**
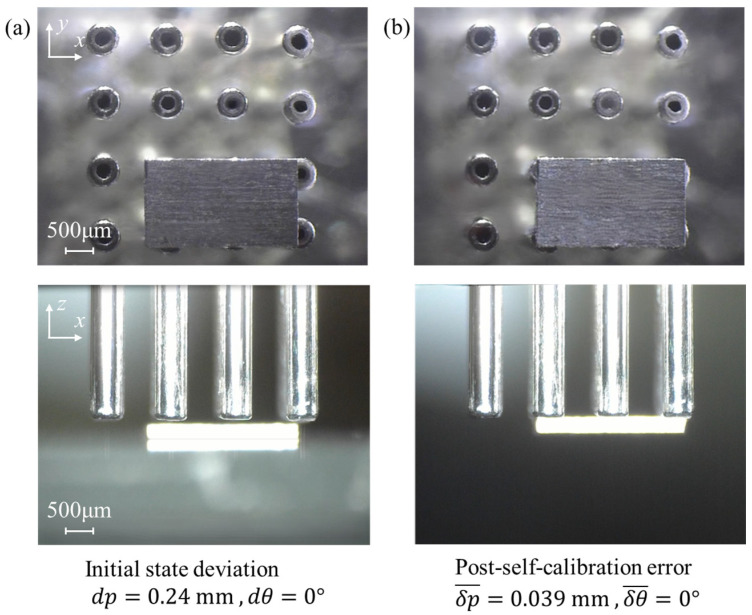
Self-calibration results of positional deviation for aluminum micro-component. The dynamic process is presented in Videos S1 and S2. (Material: Aluminum, Dimensions: 2.4 mm × 1.4 mm × 0.2 mm, Mass: 1.81 mg, Roughness Sa=1.5 μm). (**a**) Initial state deviation; (**b**) Post-self-calibration error.

Figure 12 shows the self-calibration experiment for the positional deviation of aluminum micro-components. The dimensions of the micro-component are 2.4 mm × 1.4 mm × 0.2 mm. Based on the size and shape of the micro-component, six micro-tubes at the lower right corner of the micro-tube array are selected. Distilled water is slowly injected into these micro-tubes until the droplets just wetted the operating end faces of the micro-tubes, thereby constructing the droplet substrate CS. The central positional deviation of the micro-component is denoted as dp, where $dp=\sqrt{{dx}^{2}+{dy}^{2}}$. Initially, the center position deviation between the micro-component and the calibration substrate CS is dp=0.24 mm, as shown in Figure 12a. The droplet substrate quickly picks up the micro-component upon contact, and the position and orientation of the micro-component rapidly calibrate with the CS. The center position error after self-calibration is denoted as δp, where $\delta p=\sqrt{{\delta x}^{2}+{\delta y}^{2}}$. The average value of the center position error $\bar{\delta p}$ after 10 repeated calibration experiments is 0.039 mm, as shown in Figure 12b.

**Figure 13:**
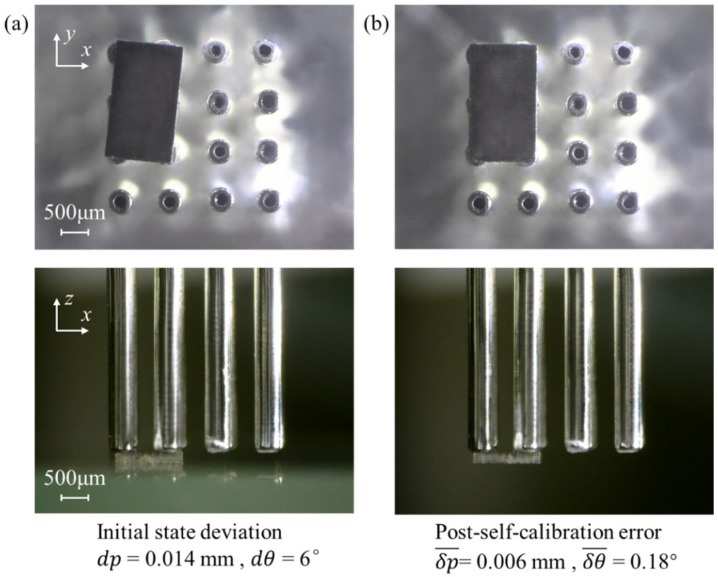
Comprehensive self-calibration results of titanium micro-component. The dynamic process is presented in Videos S3 and S4. (Material: Titanium, Dimensions: 2.4 mm × 1.4 mm × 0.2 mm, Mass: 3.02 mg, Roughness Sa=1.5 μm). (**a**) Initial state deviation; (**b**) Post-self-calibration error.

Figure 13 shows the comprehensive self-calibration experiment of the micro-component in position and orientation. The initial center position deviation between the micro-component and the calibration substrate CS is dp=0.014 mm, the orientation deviation is dθ=6°, as shown in Figure 13a. After 10 repeated self-calibration experiments, the average center position error is $\bar{\delta p}=0.006\;mm$, the average orientation error is $\bar{\delta \theta }=0.18{}^{\circ}$, as shown in Figure 13b.

**Figure 14:**
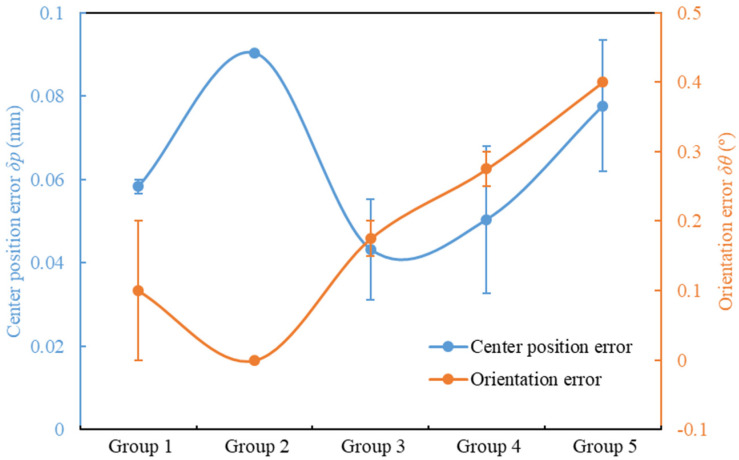
Self-Calibration Errors of the Titanium Micro-Component.

Figure 14 presents the statistical results of comprehensive self-calibration experimental errors for the titanium micro-component. Five groups of experiments with different initial deviations are designed, and each group is repeated 10 times. The initial deviation of each group is listed in Table 1. Restricted by experimental conditions, it is difficult to strictly set the initial deviation to a fixed value; therefore, the initial deviation of each group is controlled within a narrow range. It can be seen from the experimental results that for the same material, both position deviation and orientation deviation can be self-calibrated under different initial deviations. The residual error after self-calibration shows slight fluctuations, which are mainly attributed to the periodic variation in liquid bridge resistance.

**Figure 15:**
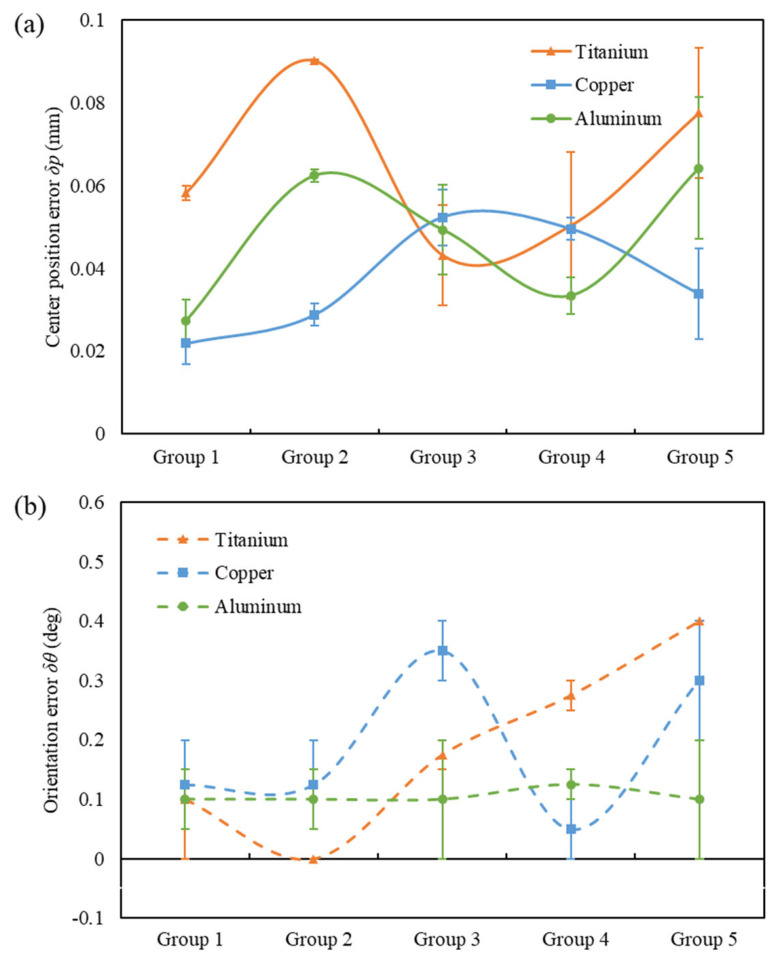
Comprehensive self-calibration errors for micro-components of three different materials with identical dimensions.

Figure 15 shows the comprehensive self-calibration experimental errors of three micro-components with identical dimensions made of titanium, copper, and aluminum, respectively. The three micro-components have the same surface roughness (Sa = 1.5 μm). Five groups of experiments with different initial deviations are designed, and each group is repeated 10 times. The initial deviation range adopted for each group is consistent with that in Table 1. Experimental results show that self-calibration can be achieved for micro-components of different materials with various initial deviations. Under the condition of identical surface roughness, the self-calibration errors are basically consistent and independent of the initial deviation of the micro-component.

### 5.3. Experiment on the Influence of Roughness

**Figure 16:**
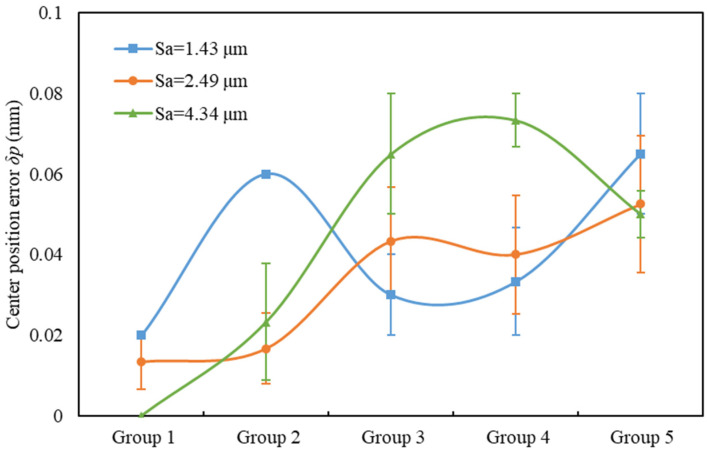
Influence of micro-component surface roughness on self-calibration error (material: aluminum, dimensions: 2.4 mm × 1.4 mm × 0.2 mm, mass: 1.81 mg).

To investigate the influence of the surface roughness of micro-components on calibration performance, self-calibration experiments were carried out using three aluminum micro-components with different roughness values. The experimental results are shown in Figure 16. Five groups of experiments were conducted, each repeated 10 times. The initial deviations for each group are shown in Table 2.

It can be seen from the figure that the self-calibration error increases with the increase in surface roughness of the metallic micro-components. This is mainly because the resistance between the liquid bridge and the surface of the micro-component rises as the roughness increases. Meanwhile, a larger initial position deviation leads to a larger final self-calibration error, which is mainly attributed to the frictional energy loss during liquid bridge sliding and the periodic variation in liquid bridge resistance.

### 5.4. Self-Calibration Experiments of Micro-Components with Different Shapes

**Figure 17:**
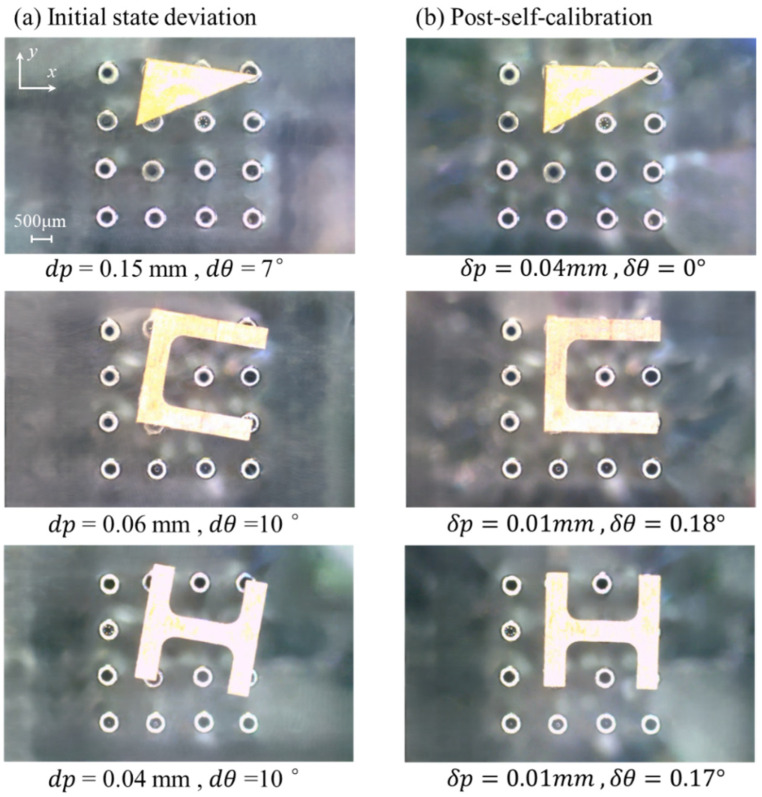
Self-calibration experiments of several irregular-shaped micro-components.

**Figure 18:**
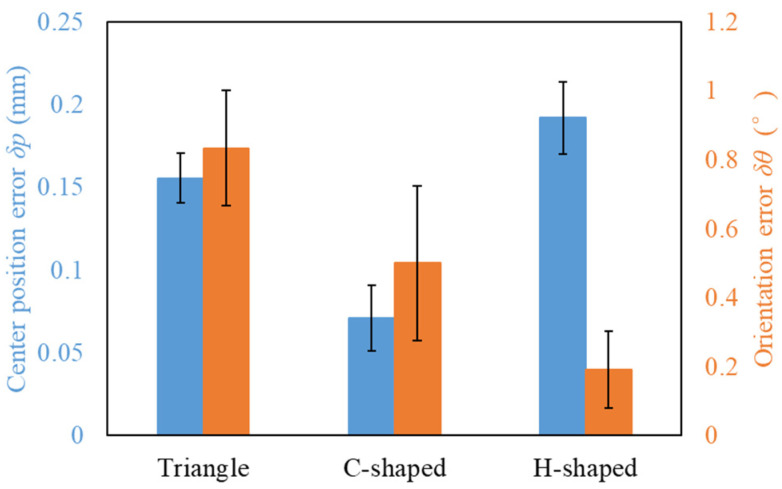
Self-calibration errors of irregular-shaped micro-components.

On the operation end surface S of the micro-tube array, droplet arrays of arbitrary shapes can be constructed according to the shape and size of the micro-component to meet the self-calibration requirements. Figure 17 shows the self-calibration experiments of several irregular copper micro-components. The initial deviations of micro-components with different shapes are approximately consistent. Each experiment was repeated six times, and the experimental results are shown in Figure 18. The experimental results demonstrate that self-calibration can be achieved for various irregular-shaped micro-components.

### 5.5. Analysis and Discussion

In industrial micro-assembly and surface-mount technology, calibration accuracy is typically evaluated using center position error and orientation error as core metrics. These indicators possess clear physical significance and facilitate horizontal comparison with industry standards. According to the IPC-A-610H standard Acceptability of Electronic Assemblies [65], the placement accuracy requirements for surface-mount components stipulate that for passive components such as 0402 and 0603 chip resistors and capacitors, the maximum allowable placement offset shall not exceed 25% of the component width or pad length. Taking the 0402 resistor (width 0.5 mm) as an example, the allowable offset is approximately ±0.1 mm (100 μm). The self-calibration center position error and orientation error obtained in the experiments of this study are significantly smaller than the allowable ranges specified in the aforementioned industrial standards, meeting the precision requirements for high-end micro-assembly and chip mounting.

Compared with the self-calibration of rectangular micro-components, irregular-shaped micro-components produce relatively larger errors in the self-calibration process. This is mainly because shape errors are introduced by different topological selections of the micro-tube array when constructing a shape-matched calibration substrate. In addition, the sharp-corner regions of irregular micro-components are hard to be fully wetted by droplets, further increasing calibration errors. Extensive experiments verify that when slight position and orientation deviations exist between micro-components and the calibration substrate, droplet substrates can realize self-calibration under complete surface wetting of micro-components. Self-calibration accuracy is affected by surface material, roughness and other factors of micro-components. Future work will establish mapping relationships between these parameters and calibration accuracy. In addition, the influence mechanisms of droplet array topological arrangement, wettability coupling and hydrodynamic interference on position-and-orientation calibration accuracy will be further investigated.

## 6. Conclusions

By utilizing the reconfigurability of the droplet array and the adaptability of the liquid bridge force, a dynamic calibration substrate is constructed using the droplet array to meet the iterative update requirements of micro-components. The construction method of the dynamic substrate is analyzed, the coupling interaction between each liquid bridge and the micro-component during the self-calibration process, as well as the self-calibration mechanism, is investigated, and the influence of micro-component parameters on the self-calibration accuracy is discussed. The research and experimental results show that:

(1) According to the parameters and target position and orientation of the micro-component, the corresponding calibration substrate CS is constructed, which enables the automatic self-calibration of wettable micro-components. The self-calibration accuracy results from the combined action of all liquid bridges on the micro-component and is related to the surface material and surface roughness of the micro-component. For a given micro-component material, the calibration accuracy depends on the surface roughness: the smoother the micro-component surface, the smaller the resistance exerted by the liquid bridge, and the higher the calibration accuracy. Different micro-component materials lead to different contact angles at the liquid–solid interface. For cases with similar contact angles, self-calibration can be achieved with basically consistent errors.
(2) Taking a 4 × 4 micro-tube array as an example, this paper illustrates the construction method of the self-calibration platform. The proposed method is also applicable to the construction of m × n micro-tube arrays. Through a switch array, the connection between the micro-tube array and the droplet generation device can be controlled, generating droplet arrays of different shapes, sizes, and numbers to meet the calibration requirements of micro-components with different parameters.
(3) Multiple calibration substrates, CS or CS with different shapes and sizes, can be constructed on an m × n micro-tube array, which enables high-throughput self-calibration of the same type of micro-components and simultaneous calibration of micro-components with different shapes, satisfying the demands of high-efficiency, high-throughput, and high-adaptability calibration.

## Figures and Tables

**Table 1:** Initial Deviations in the Self-Calibration Experiment of the Titanium Micro-Component.

Experimental Group Number	Initial Central Position Deviation dp (mm)	Initial Orientation Deviation dθ (°)
1	0.09–0.12	1–3°
2	0.12–0.15	3–5°
3	0.15–0.18	5–7°
4	0.18–0.21	7–9°
5	0.21–0.24	9–11°

**Table 2:** Initial central position deviation *dp* (mm) for self-calibration of aluminum micro-components with three different roughness levels.

Experimental Group Number	Initial Central Position Deviation dp (mm)
1	0.10–0.13
2	0.13–0.16
3	0.16–0.19
4	0.19–0.22
5	0.22–0.25

## Data Availability

The original contributions presented in this study are included in the article. Further inquiries can be directed to the corresponding author.

## Supplementary Materials

The following supporting information can be downloaded at: https://www.mdpi.com/article/10.3390/mi17060669/s1. Video S1. Self-calibration results of positional deviation for aluminum micro-component-Top view. Video S2. Self-calibration results of positional deviation for aluminum micro-component-Front view. Video S3. Comprehensive self-calibration results of titanium micro-component-Top view. Video S4. Comprehensive self-calibration results of titanium micro-component-Front view.

## References

[1] Tanaka K., Ito T., Nishiyama Y., Fukuchi E., Fuchiwaki O. (2022). Double-Nozzle Capillary Force Gripper for Cubical, Triangular Prismatic, and Helical 1-mm-Sized-Objects. IEEE Robot. Autom. Lett..

[2] Ito T., Fukuchi E., Tanaka K., Nishiyama Y., Watanabe N., Fuchiwaki O. (2022). Vision Feedback Control for the Automation of the Pick-and-Place of a Capillary Force Gripper. Micromachines.

[3] Kim K., Liu X.Y., Zhang Y., Sun Y. (2008). Nanonewton Force-Controlled Manipulation of Biological Cells Using a Monolithic MEMS Microgripper with Two-Axis Force Feedback. J. Micromech. Microeng..

[4] Chen B.K., Zhang Y., Sun Y. (2009). Active Release of Microobjects Using a MEMS Microgripper to Overcome Adhesion Forces. J. Microelectromech. Syst..

[5] Zhang Y., Chen B.K., Liu X.Y., Sun Y. (2010). Autonomous Robotic Pick-and-Place of Microobjects. IEEE Trans. Robot..

[6] Liu S., Xu D., Liu F.F., Zhang D.P., Zhang Z.T. (2016). Relative Pose Estimation for Alignment of Long Cylindrical Components Based on Microscopic Vision. IEEE/ASME Trans. Mechatron..

[7] Liu S., Xu D., Zhang D.P., Zhang Z.T. (2016). High Precision Automatic Assembly Based on Microscopic Vision and Force Information. IEEE Trans. Autom. Sci. Eng..

[8] Tang Y.L., Zhang Z.J., Ye X., Zhang X.F. (2014). Micro-Assembly Precise Coaxial Alignment Methodology Based on Surface Roughness and Reflectiveness Matching. Assem. Autom..

[9] Li A., Li H., Li Z., Zhao Z., Song Y. (2020). Programmable Droplet Manipulation by a Magnetic-Actuated Robot. Sci. Adv..

[10] Lin J.L., Hsu P.P., Kuo J.N. (2023). Magnetic Beads inside Droplets for Agitation and Splitting Manipulation by Utilizing a Magnetically Actuated Platform. Micromachines.

[11] Ramadan Q., Uk Y.S., Vaidyanathan K. (2007). Large Scale Microcomponents Assembly Using an External Magnetic Array. Appl. Phys. Lett..

[12] Shet S., Mehta V.R., Fiory A.T., Lepselter M.P., Ravindra N.M. (2004). The magnetic field-assisted assembly of nanoscale semiconductor devices: A new technique. JOM.

[13] Zhuang J.J., Lin S., Dong L.Q., Cheng K., Weng W.J. (2018). Magnetically Assisted Electrodeposition of Aligned Collagen Coatings. ACS Biomater. Sci. Eng..

[14] Cha H., Ouyang L., Chen X., Wu Y., Kang X., An H., Li W., Nguyen N.T., Zhang J. (2025). Leveraging Dielectrophoresis in Inertial Flow for Versatile Manipulation of Micro and Nanoparticles. Lab Chip.

[15] Lee S.W., Bashir R. (2003). Dielectrophoresis and Electrohydrodynamics-Mediated Fluidic Assembly of Silicon Resistors. Appl. Phys. Lett..

[16] Xu W., Jin Y., Li W., Song Y., Gao S., Zhang B., Wang L., Cui M., Yan X., Wang Z. (2022). Triboelectric Wetting for Continuous Droplet Transport. Sci. Adv..

[17] Li J., Ha N.S., Liu T.L., Van Dam R.M., Kim C.J.C. (2019). Ionic-surfactant-mediated electro-dewetting for digital microfluidics. Nature.

[18] Ye W.Q., Zhang W., Xu Z.R. (2024). Shape-memory microfluidic chips for fluid and droplet manipulation. Biomicrofluidics.

[19] Ke X., Shen X., Li T., Duan X. (2026). Droplet acoustofluidics: From acoustic principles to micro manipulations. Nanotechnol. Precis. Eng..

[20] Chang B., Feng Y.H., Jin J.L., Zhou Q. (2021). Ejected Droplet-Directed Transportation and Self-Alignment of Microfibers to Micro Trenches. J. Microelectromech. Syst..

[21] Chang B., Jin J., Zhou Q. (2020). Surface Tension-Based Alignment of Microfibers on Hydrophilic–Superhydrophobic Grooved Surfaces. Micromachines.

[22] Chang B., Liu H., Ras R.H.A., Zhou Q. (2019). Capillary Transport of Miniature Soft Ribbons. Micromachines.

[23] Chang B., Routa I., Sariola V., Zhou Q. (2011). Self-alignment of RFID Dies on Four-pad Patterns with Water Droplet for Sparse Self-assembly. J. Micromech. Microeng..

[24] Chang B., Shah A., Routa I., Lipsanen H., Zhou Q. (2012). Surface-tension Driven Self-assembly of Microchips on Hydrophobic Receptor Sites with Water Using Forced Wetting. Appl. Phys. Lett..

[25] Chang B., Zhou Q., Wu Z.G., Liu Z.H., Ras R.H.A., Hjort K. (2016). Capillary Self-Alignment of Microchips on Soft Substrates. Micromachines.

[26] Chang B., Zhu Z.F., Koverola M., Zhou Q. (2017). Laser-Assisted Mist Capillary Self-Alignment. Micromachines.

[27] Fukushima T., Iwata E., Konno T., Bea J.C., Lee K.W., Tanaka T., Koyanagi M. (2010). Surface Tension-Driven Chip Self-Assembly with Load-Free Hydrogen Fluoride-Assisted Direct Bonding at Room Temperature for Three-Dimensional Integrated Circuits. Appl. Phys. Lett..

[28] Fukushima T., Konno T., Iwata E., Kobayashi R., Kojima T., Murugesan M., Bea J.C., Lee K.W., Tanaka T., Koyanagi M. (2011). Self-Assembly of Chip-Size Components with Cavity Structures: High-Precision Alignment and Direct Bonding without Thermal Compression for Hetero Integration. Micromachines.

[29] Sariola V., Jaaskelainen M., Zhou Q.A. (2010). Hybrid Microassembly Combining Robotics and Water Droplet Self-Alignment. IEEE Trans. Robot..

[30] Scott K.L., Hirano T., Yang H., Howe R.T., Niknejad A.M. (2004). High-Performance Inductors Using Capillary Based Fluidic Self-Assembly. J. Microelectromech. Syst..

[31] Xiaorong X., Hanein Y., Jiandong F., Yanbing W., Weihua W., Schwartz D.T., Bohringer K.F. (2003). Controlled Multibatch Self-Assembly of Microdevices. J. Microelectromech. Syst..

[32] Bohringer K.F., Srinivasan U., Howe R.T. (2001). Modeling of Capillary Forces and Binding Sites for Fluidic Self-Assembly. Proceedings of the Technical Digest, MEMS 2001, 14th IEEE International Conference on Micro Electro Mechanical Systems (Cat. No.01CH37090), Interlaken, Switzerland, 21–25 January 2001.

[33] Srinivasan U., Liepmann D., Howe R.T. (2001). Microstructure to Substrate Self-Assembly Using Capillary Forces. J. Microelectromech. Syst..

[34] Kaltwasser M., Schmidt U., Biswas S., Reiprich J., Schlag L., Isaac N.A., Stauden T., Jacobs H.O. (2018). Core-Shell Transformation-Imprinted Solder Bumps Enabling Low-Temperature Fluidic Self-Assembly and Self-Alignment of Chips and High Melting Point Interconnects. ACS Appl. Mater. Interfaces.

[35] Chang B., Shah A., Zhou Q., Ras R.H.A., Hjort K. (2015). Self-Transport and Self-Alignment of Microchips Using Microscopic Rain. Sci. Rep..

[36] Stauth S.A., Parviz B.A. (2006). Self-Assembled Single-Crystal Silicon Circuits on Plastic. Proc. Natl. Acad. Sci. USA.

[37] Tsai C.G., Hsieh C.M., Yeh J.A. (2007). Self-Alignment of Microchips Using Surface Tension and Solid Edge. Sens. Actuators A Phys..

[38] Weinstein T., Gilon H., Filc O., Sammartino C., Pinchasik B.-E. (2022). Automated Manipulation of Miniature Objects Underwater Using Air Capillary Bridges: Pick-and-Place, Surface Cleaning, and Underwater Origami. ACS Appl. Mater. Interfaces.

[39] Constante G., Apsite I., Auerbach P., Aland S., Schönfeld D., Pretsch T., Milkin P., Ionov L. (2022). Smart Mechanically Tunable Surfaces with Shape Memory Behavior and Wetting-Programmable Topography. ACS Appl. Mater. Interfaces.

[40] Guo C., Pan Z.X., Li C.H., Zou S.H., Pang C., Wang J.T., Hu J.H., Gong Z. (2022). Large-scale Programmable Assembly of Functional Micro-components for Advanced Electronics via Light-regulated Adhesion and Polymer Growth. npj Flex. Electron..

[41] Zhang L., Zhang L.L., Xu J.Y., Luo Y.F., Liu J.H., Wu T.C., Hao B. (2025). Shape Memory Polymer Microtransfer Printing Stamp with Macro-Micro Adjustable Adhesion Superhydrophobic Surface Obtained by Laser Texturing. ACS Appl. Mater. Interfaces.

[42] Jeske M.P., Wang H.N., Askari H., Harding D.R., Anthamatten M. (2025). Adhesion of Self-Complementary, Sinusoidal Surfaces Fabricated Using Two-Photon Polymerization. ACS Appl. Polym. Mater..

[43] Wang D., Jin K., Ji J., Hu C., Du M., Belgaid Y., Shi S., Li J., Hu S., Nathan A. (2024). Active-Matrix Digital Microfluidics Design for Field Programmable High-throughput Digitalized Liquid Handling. iScience.

[44] Wang T., Zhou S., Liu X., Zeng J., He X., Yu Z., Liu Z., Liu X., Jin J., Zhu Y. (2025). Intelligent Optoelectrowetting Digital Microfluidic System for Real-time Selective Parallel Manipulation of Biological Droplet Arrays. Lab Chip.

[45] Wu S.Z., Li D.Y., Zhang J., Zhang Y.Y., Zhang Y.X., Li S.Y., Chen C., Guo S.J., Li C.Z., Lao Z.X. (2023). Multiple-Droplet Selective Manipulation Enabled by Laser-Textured Hydrophobic Magnetism-Responsive Slanted Micropillar Arrays with an Ultrafast Reconfiguration Rate. Langmuir.

[46] Melin J., Quake S.R. (2009). Microfluidic large-scale integration: The evolution of design rules for biological automation. Annu. Rev. Biophys. Biomol. Struct..

[47] Unger M.A. (2000). Monolithic Microfabricated Valves and Pumps by Multilayer Soft Lithography. Science.

[48] Thorsen T., Maerkl S.J., Quake S.R. (2002). Microfluidic Large-Scale Integration. Science.

[49] Brouzes E., Medkova M., Savenelli N., Marran D., Twardowski M., Hutchison J.B., Rothberg J.M., Link D.R., Perrimon N., Samuels M.L. (2009). Droplet microfluidic technology for single-cell high-throughput screening. Proc. Natl. Acad. Sci. USA.

[50] Raveshi M.R., Halim M.S.A., Agnihotri S.N., O’Bryan M.K., Neild A., Nosrati R. (2021). Curvature in the reproductive tract alters sperm–surface interactions. Nat. Commun..

[51] Vafaie A., Shahali S., Raveshi M.R., Nosrati R., Neild A. (2025). Repeated pulses of ultrasound maintain sperm motility. Lab Chip.

[52] Zhu P., Wang L. (2016). Passive and active droplet generation with microfluidics: A review. Lab Chip.

[53] Huang C., Jiang Y., Li Y., Zhang H. (2022). Droplet Detection and Sorting System in Microfluidics: A Review. Micromachines.

[54] Agnihotri S.N., Raveshi M.R., Nosrati R., Bhardwaj R., Neild A. (2025). Droplet splitting in microfluidics: A review Open Access. Phys. Fluids.

[55] Teh S.Y., Lin R., Hung L.H., Lee A.P. (2008). Droplet microfluidics. Lab Chip.

[56] Yan H., Qin Z., Ciwen M., Zhiming O. (2022). Self-calibration of micro-components based on manipulator guidance. J. Micromech. Microeng..

[57] Qin Z., Shen J., Yan H. (2022). Method and experiment of self-calibration micro-components based on droplet array. Proceedings of the 2022 IEEE International Conference on Robotics and Biomimetics (ROBIO), Jinghong, China, 5–9 December 2022.

[58] Yan H., Qin Z., Shen J. (2024). Self-Calibration Performance Analysis of Sheet Micro-Components Based on Droplet Array. J. Phys. Conf. Ser..

[59] Kaminski T.S., Garstecki P. (2017). Controlled droplet microfluidic systems for multistep chemical and biological assays. Chem. Soc. Rev..

[60] Agnihotri S.N., Raveshi M.R., Bhardwaj R., Neild A. (2021). Microvalves for integrated selective droplet generation, splitting and merging on a chip. Microfluid. Nanofluid..

[61] Lambert P. (2013). Surface Tension in Microsystems.

[62] Wang X.D., Peng X.F., Min J.C., Liu T. (2001). Hysteresis of contact angle at liquid-solid interface. J. Basic Sci. Eng..

[63] Iliev S., Pesheva N., Iliev P. (2023). Contact angle hysteresis on random self-affine rough surfaces in Wenzel’s wetting regime: Numerical study. Phys. Rev. E.

[64] Qingrui S. (2023). Study on the Adhesion Mechanics of a Liquid Bridge Under Shear. Doctoral Dissertation.

[65] (2020). Acceptability of Electronic Assemblies.

